# Endobronchial Hamartoma: A Retrospective Cohort Study of 17 Cases and Systematic Review of the Contemporary Literature

**DOI:** 10.3390/jcm15103616

**Published:** 2026-05-08

**Authors:** Qianqian Hua, Xiaoyan Chen, Wei Chen, Yi Guo

**Affiliations:** 1 Department of Pulmonary and Critical Care Medicine, Ruijin Hospital, Shanghai Jiao Tong University School of Medicine, Shanghai 200025, China; hqq01q31@rjh.com.cn (Q.H.); cxy11832@rjh.com.cn (X.C.); cw11242@rjh.com.cn (W.C.); 2 Institute of Respiratory Diseases, Shanghai Jiao Tong University School of Medicine, Shanghai 200025, China

**Keywords:** endobronchial hamartoma, bronchoscopy, surgery, recurrence, lung preservation

## Abstract

**Background:** Endobronchial hamartoma (EBH) is an exceptionally rare benign neoplasm frequently misdiagnosed as an obstructive malignancy. The therapeutic paradigm is shifting from traditional anatomical resection toward parenchyma-preserving interventional techniques. This study evaluates the efficacy and safety profiles of contemporary bronchoscopic interventions versus surgical management for EBH. **Methods:** A retrospective analysis was conducted on a clinical cohort of 17 patients treated between 2013 and 2026, alongside a comprehensive systematic review of 31 contemporary studies (2013–2025). The primary endpoint was the treatment success rate at 3 months, while secondary outcomes included perioperative complications, re-intervention rates, and successful lung parenchyma preservation. **Results:** Within the analyzed cohort (median age, 58 years), lesions exhibited a significant right-sided predilection (70.6%). Preoperative imaging uniformly revealed non-specific masses, with 41.2% displaying secondary obstructive manifestations. Definitive interventions comprised bronchoscopic management (*n* = 11, 64.7%) and surgical resection (*n* = 6, 35.3%). The technical success rate was 100%, with zero major perioperative complications and only minimal-to-scant intraoperative bleeding reported. Over a median follow-up of 3 months, local recurrence was observed in three cases (17.6%)—notably spanning both surgical (*n* = 2) and bronchoscopic (*n* = 1) modalities. The systematic review corroborated these findings, underscoring the exemplary safety profile and superior lung-sparing capacity of bronchoscopic interventions. **Conclusions:** Within the limits of this retrospective cohort and literature review, interventional bronchoscopy appears to be a safe and lung-sparing approach. It may be considered as a preferable initial treatment option for anatomically suitable EBHs. Traditional surgical resection remains necessary for anatomically complex lesions or cases with irreversible distal parenchymal destruction. Vigilant longitudinal surveillance is advised across all modalities.

## 1. Introduction

Endobronchial hamartoma (EBH) is an exceptionally rare benign neoplasm of the central airways. Although pulmonary hamartomas as a whole constitute the vast majority, approximately 75% to 77% of all benign lung tumors, their endobronchial variant is markedly uncommon, accounting for barely 1% to 1.5% of all benign pulmonary neoplasms [1]. Given its remarkably low incidence compared to malignant endobronchial lesions, EBH is frequently overlooked or misdiagnosed in clinical practice, often mimicking central lung cancer, foreign body aspiration, or chronic inflammatory stenosis [2]. Histologically, the lesion is defined by a disorganized proliferation of mature mesenchymal elements-predominantly cartilage, adipose tissue, and fibrous stroma-interspersed with normal respiratory epithelium. Despite its benign pathological nature, the progressive intraluminal growth of EBH provokes severe mechanical airflow limitation and distal secretion retention. Consequently, patients typically present with a spectrum of obstructive symptoms, including chronic cough, wheezing, and hemoptysis, which can ultimately culminate in recurrent obstructive pneumonia, atelectasis, and irreversible parenchymal damage if left untreated [3,4].

Historically, the management of EBH primarily relied on surgical resection, including bronchotomy, segmentectomy, or lobectomy; more extensive surgeries were often necessitated by delayed diagnosis or significant distal lung destruction [5]. In recent years, however, the advent of pulmonary interventional procedures, incorporating bronchoscopic techniques such as snare electroresection, thermal ablation (electrocautery, laser, argon plasma coagulation), and cryotherapy, has enabled the minimally invasive excision of intraluminal lesions while maximizing parenchymal preservation. This paradigm shift has transitioned clinical decision making from a binary assessment of “resectability” toward a more nuanced selection of the optimal therapeutic modality to ensure both efficacy and safety.

Owing to its extreme rarity, the current body of literature concerning EBH is predominantly limited to isolated case reports and fragmented case series. Consequently, substantial evidence gaps persist regarding its clinical phenotypes, radiologic and bronchoscopic characteristics, optimal therapeutic algorithms, and long-term prognostic outcomes [6]. To bridge these knowledge gaps, we designed a complementary dual-methodology study. First, we retrospectively analyzed a highly granular, 13-year institutional cohort to investigate precise clinicopathological features and procedural outcomes. Second, to overcome the inherent limitations of a small, single-center sample, we integrated a systematic review of the contemporary global literature (2013–2025). Specifically, this study aims to: (1) systematically delineate the baseline characteristics, interventional modalities, and longitudinal outcomes of the cohort; (2) conduct a comprehensive review of the existing literature on this therapeutic paradigm; and (3) synthesize our institutional experience with global data to propose a robust, evidence-based framework for the clinical management of this rare airway neoplasm.

## 2. Methods

### 2.1. Overall Study Design and Integration Strategy

This research employs a dual-design framework consisting of an institutional retrospective cohort integrated with a PRISMA-compliant systematic review (File S1). The institutional cohort was utilized to extract granular procedural and perioperative data, while the systematic review was designed to aggregate global clinical outcomes to validate the local findings and extend the observational timeframe. The data extraction variables for the systematic review were explicitly aligned with the cohort endpoints to ensure seamless synthesis during the discussion.

### 2.2. Diagnostic Biopsy Strategy and Treatment Selection Criteria

Because EBH often mimics central airway malignancy, our institutional protocol mandates a pathological evaluation prior to definitive therapeutic intervention. Pre-treatment biopsies were predominantly obtained via flexible bronchoscopy utilizing forceps or transbronchial cryobiopsy. In specific instances where the lesion was predominantly peripheral, CT-guided percutaneous needle biopsy (PNB) was utilized.

Furthermore, the selection of definitive treatment was non-randomized and driven by multidisciplinary anatomical assessment. Bronchoscopic intervention was prioritized for lesions that were primarily intraluminal, pedunculated, or possessed a narrow base. Conversely, traditional surgical resection was strictly indicated for lesions exhibiting broad-based transmural invasion, or when chronic obstruction had resulted in irreversible distal lung parenchymal destruction.

### 2.3. Study Population and Data Collection

**Figure 1:**
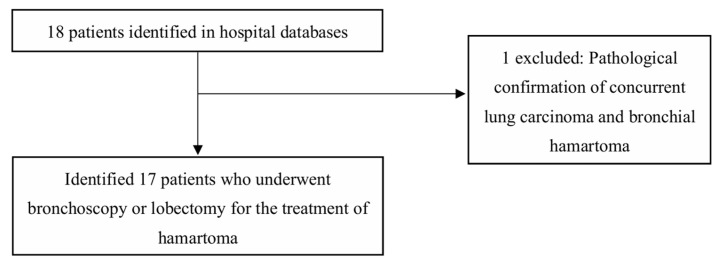
Flowchart of patient recruitment and selection. A total of 18 patients with a potential diagnosis of hamartoma are initially identified from the hospital database. One patient is excluded due to parenchymal pulmonary hamartoma without endobronchial involvement. Consequently, 17 patients who met the criteria and underwent therapeutic interventions for endobronchial hamartoma (EBH) are included in the final retrospective analysis.

This retrospective study analyzed a clinical cohort of patients pathologically diagnosed with EBH at Ruijin Hospital, Shanghai Jiao Tong University School of Medicine, between January 2013 and January 2026. Inclusion criteria required a pathologically confirmed EBH diagnosis with comprehensive clinical, radiological, and follow-up records. Patients were excluded if they underwent diagnostic procedures without therapeutic intent, lacked valid follow-up data, or had incomplete medical records. Clinical data were independently extracted from electronic medical records by two investigators—with discrepancies resolved by a third senior reviewer, capturing baseline demographics, symptomatology, radiologic and bronchoscopic features, procedural details, and pathological outcomes (Figure 1). The Ethics Committee of Ruijin Hospital, Shanghai Jiao Tong University School of Medicine, approved this study (Approval n° 2023–199). All participants provided informed consent before enrollment. https://clinicaltrials.gov/ct2/show/NCT06082791 (accessed on 1 February 2026). This study was reported in accordance with the STROBE (Strengthening the Reporting of Observational Studies in Epidemiology) statement guidelines for observational studies.

### 2.4. Outcome Measures

The primary outcome was defined as the treatment success rate at 3 months post-intervention. In accordance with the established interventional pulmonology literature, this 3-month timeframe serves as the critical clinical boundary for evaluating complete mucosal restitution, early procedural thoroughness, and the absence of immediate residual disease following endobronchial resection [7,8]. This was characterized by the complete macroscopic eradication of the lesion and the resolution of baseline symptoms, without clinical or bronchoscopic evidence of recurrence requiring further intervention at the 3-month follow-up mark. Secondary endpoints included the standardized 30-day perioperative complication rate (encompassing any procedure-related adverse events) [9,10], the overall re-intervention rate for residual or recurrent disease [11].

### 2.5. Literature Review: Search Strategy and Selection

**Figure 2:**
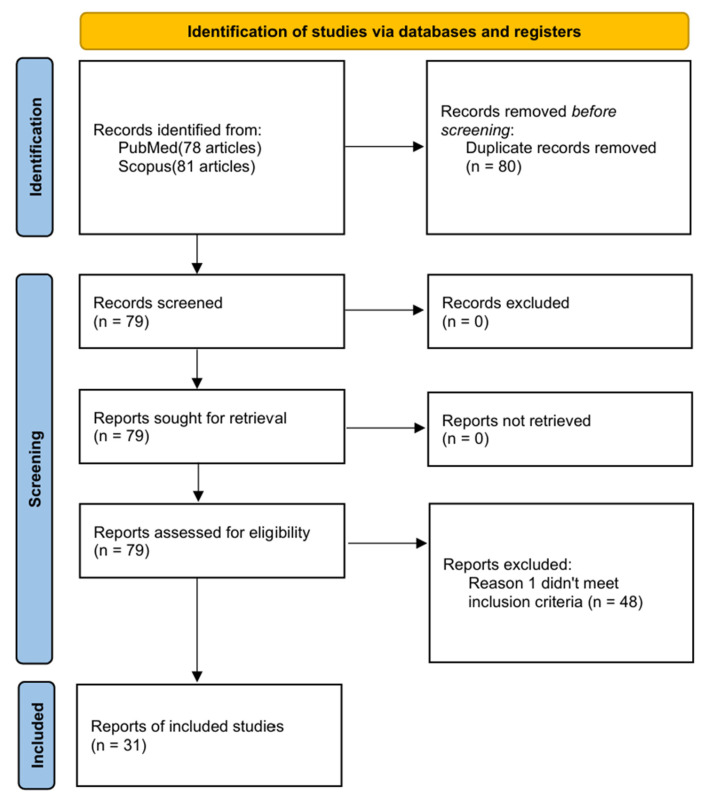
PRISMA flowchart detailing the literature search and study selection process. A total of 159 records are identified through database searching (PubMed: *n* = 78; Scopus: *n* = 81). After the removal of 80 duplicate records, 79 titles and abstracts are screened. No records are excluded during the initial screening. Upon full-text assessment of the 79 reports, 48 are excluded for not meeting the specific inclusion criteria. Finally, 31 studies are included in the systematic review.

Following PRISMA guidelines (Figure 2), a systematic literature search of PubMed and Scopus was conducted on 1 March 2026. To ensure a comprehensive capture of the literature, we employed a structured Boolean search strategy combining MeSH terms and free-text keywords. The primary search string utilized was: ((“Endobronchial hamartoma” [Title/Abstract] OR “Bronchial hamartoma” [Title/Abstract]” AND (“Bronchoscopy” [Title/Abstract] OR “Intervention” [Title/Abstract] OR “Surgery” [Title/Abstract] OR “Resection” [Title/Abstract] OR “Endoscopic” [Title/Abstract])) to identify relevant English-language original studies, case series, and case reports published between 2013 and 2025. Two independent reviewers screened abstracts and performed full-text evaluations to isolate confirmed cases with accessible full-text data, resolving any discrepancies via consensus. Finally, data concerning clinical outcomes, epidemiological parameters, and study frameworks were extracted to align with our institutional cohort.

### 2.6. Statistical Analysis

Descriptive statistics were employed to summarize the anonymized patient cohort. All statistical analyses were performed using R software (version 4.3.2; R Foundation for Statistical Computing, Vienna, Austria). Given the descriptive nature of this study, no inter-group comparisons or hypothesis testing were conducted. Categorical variables are strictly expressed as frequencies (n) and percentages (%). Where applicable, continuous variables are reported as medians with corresponding ranges to accurately reflect the baseline clinical characteristics.

Because the existing literature on EBH is inherently restricted to case reports and small observational case series lacking control cohorts, a formal quantitative meta-analysis was methodologically precluded. Instead, a structured qualitative synthesis (narrative synthesis) was conducted. Clinical outcomes from the systematic review were systematically stratified and synthesized according to three key dimensions: (1) primary therapeutic modality (flexible bronchoscopy vs. rigid bronchoscopy vs. surgical resection), (2) perioperative complication profiles, and (3) longitudinal recurrence patterns. This structured framework allowed for a direct, comparative integration with our institutional cohort data.

Clinical heterogeneity was qualitatively explored by stratifying the included studies according to three key dimensions: primary therapeutic modality (bronchoscopic intervention vs. surgical resection vs. conservative management) and follow-up duration (<12 months vs. ≥12 months). The impact of these differences on treatment outcomes was discussed in the Section 3 and Section 4.

### 2.7. Risk of Bias Assessment

The methodological quality of included case reports and case series was independently assessed by two reviewers using the Joanna Briggs Institute (JBI) Critical Appraisal Checklist for Case Reports and Case Series. Discrepancies were resolved by consensus with a third senior reviewer. The checklist for case reports included 8 domains: patient demographics, clinical history, clinical findings, diagnostic assessments, treatment interventions, post-intervention outcomes, adverse events, and takeaway lessons. The checklist for case series included 10 domains: study design, study population, clinical characteristics, diagnostic criteria, interventions, outcomes, follow-up, statistical analysis, adverse events, and conclusions. Each item was scored as “Yes”, “No”, “Unclear”, or “Not applicable”. Studies with a score ≥70% were considered to have low risk of bias, 50–69% moderate risk, and <50% high risk.

## 3. Results

### 3.1. Baseline Characteristics and Radiological Manifestations of Cases from Our Hospital

Baseline demographic and clinical characteristics of all 17 patients are summarized in Table 1. The final analysis comprised 17 cases, demonstrating a slight female predominance (*n* = 10, 58.8%) with a median age of 58 years (range: 26–88 years). The cohort consisted predominantly of non-smokers (*n* = 16, 94.1%), and nearly half of the patients (*n* = 8, 47.1%) had no underlying comorbidities. Clinically, 15 patients (88.2%) were symptomatic at presentation. The remaining two cases (11.8%) were entirely asymptomatic and discovered incidentally. Among the symptomatic cohort, cough was the most prevalent chief complaint (*n* = 12, 70.6%), frequently accompanied by expectoration (*n* = 7, 41.2%), chest tightness or dyspnea (*n* = 4, 23.5%), and chest or back pain (*n* = 4, 23.5%).

Anatomically, the lesions exhibited a significant predilection for the right bronchial tree (*n* = 12, 70.6%). The right lower lobe bronchus was the most frequently involved site (*n* = 5, 29.4%), followed by the right middle lobe (*n* = 3, 17.6%), right upper lobe (*n* = 3, 17.6%), and right main bronchus (*n* = 1, 5.9%). Left airway involvement was observed in five cases (29.4%). The median maximum tumor diameter was 1.6 cm (range: 0.3–7.5 cm).

**Figure 3:**
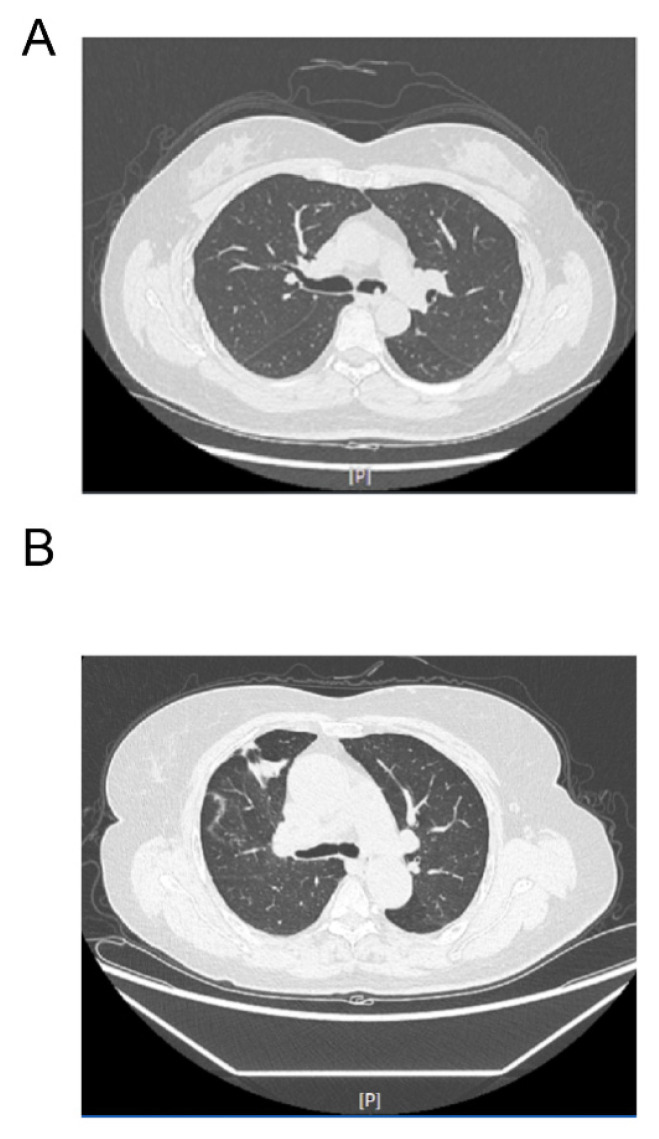
Representative thoracic CT images showing endobronchial lesions and secondary pulmonary changes: (**A**) Axial contrast-enhanced CT image demonstrating an intraluminal polypoid lesion within the airway, with associated peripheral secondary inflammatory consolidation in the right lung. (**B**) Axial CT image demonstrating an endobronchial mass causing localized obstruction in the right bronchial tree. (window settings: [P], pulmonary window).

Radiologically, preoperative contrast-enhanced CT scans uniformly revealed non-specific soft-tissue density masses, predominantly round or oval, lacking pathognomonic features. Secondary radiological manifestations were identified in seven cases (41.2%), comprising pneumothorax (*n* = 2, 11.8%), obstructive pneumonia or pulmonary infection (*n* = 2, 11.8%), pleural effusion (*n* = 1, 5.9%), pulmonary pneumatocele (*n* = 1, 5.9%), and an inflammatory nodule (*n* = 1, 5.9%). Under bronchoscopic visualization, tumor morphology was heterogeneous. Identifiable macroscopic patterns included smooth-surfaced pedunculated masses (*n* = 3, 17.6%), broad-based smooth-surfaced lesions (*n* = 3, 17.6%), and a cauliflower-like neoplasm (*n* = 1, 5.9%), while the remaining lesions presented with non-specific endobronchial appearances (*n* = 10, 58.8%) (Figure 3). Radiological and bronchoscopic characteristics of all lesions are detailed in Table 2.

### 3.2. Implementation of Therapeutic Interventions and Perioperative Safety

**Figure 4:**
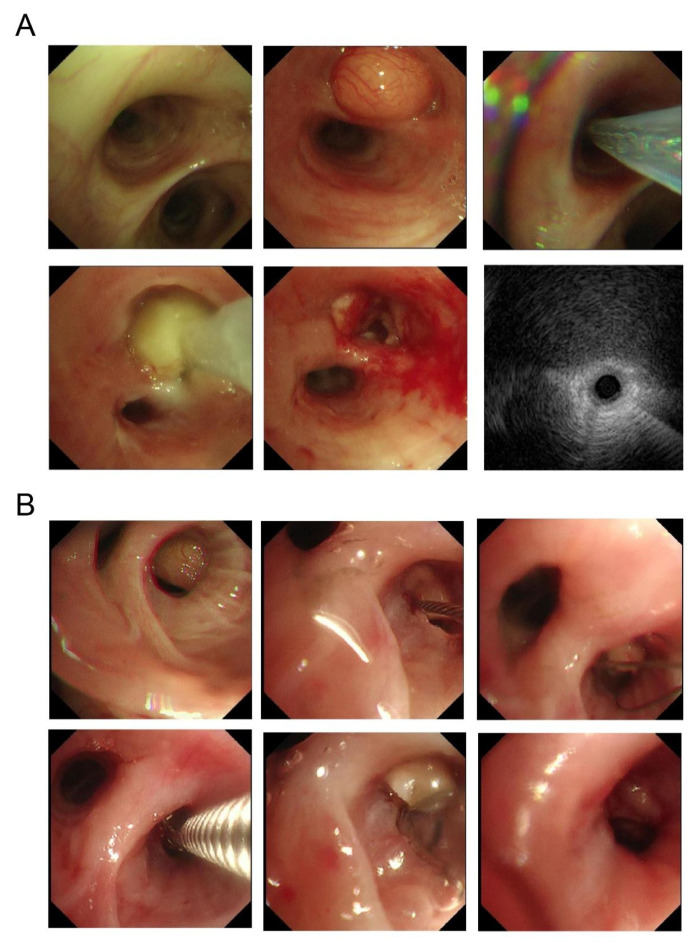
Representative bronchoscopic findings and interventional procedures in two patients with endobronchial hamartoma: (**A**) Patient 1: (1) overview of the right intermediate bronchus; (2) visualization of a neoplasm at the orifice of the right middle lobe (RML); (3) resection of the lesion using an electrosurgical snare; (4) immediate post-procedural view showing a patent RML orifice; (5) radial endobronchial ultrasound (R-EBUS) showing a well-defined lesion with heterogeneous internal echogenicity. (**B**) Patient 2: (1) identification of an endobronchial lesion in the posterior segmental sub-branch of the right upper lobe (RUL); (2) snaring of the RUL lesion; (3) retrieval of the tumor using an endoscopic basket; (4) conventional forceps biopsy; (5) transbronchial cryobiopsy (TBCB); (6) post-intervention status showing successful debulking.

All 17 patients successfully underwent definitive treatment. According to the primary therapeutic modality, patients were categorized into a bronchoscopic intervention group (*n* = 11, 64.7%), treated with electrosurgical snare resection with or without adjunctive cryoablation, and a surgical intervention group (*n* = 6, 35.3%), consisting of five lobectomies and one mass enucleation. Prior to definitive treatment, diagnostic percutaneous needle biopsy (PNB) was performed in five cases (29.4%). Detailed information on therapeutic interventions, pathological findings, and clinical outcomes is presented in Table 3. In the bronchoscopic group, cryoablation was additionally applied in three cases (Cases 1, 2, and 4) to optimize tumor debulking and margin control (Figure 4). Technical success was achieved in all cases, and postoperative histopathological examination confirmed the diagnosis of hamartoma or chondroid hamartoma in every patient.

The overall perioperative safety profile was acceptable. No major complications, including life-threatening hemoptysis, airway perforation, or procedure-related mortality, were encountered during the study period. Both bronchoscopic and surgical procedures were completed without severe treatment-related adverse events.

### 3.3. Follow-Up and Recurrence Analysis

After the initial treatment, three cases (17.6%) showed local recurrence or required additional management during subsequent clinical follow-up. These cases were observed across different treatment modalities, including one after bronchoscopic snare resection combined with cryoablation (Case 1) and two after surgical lobectomy (Cases 5 and 14). The current retrospective review of the operative and bronchoscopic records for the three recurrent cases (Cases 1, 5, and 14) revealed a shared anatomical characteristic: deep mural involvement with microscopic submucosal extension beyond the macroscopic tumor margins. In Case 1, despite intraluminal eradication, deep cartilaginous roots within the bronchial wall led to local regrowth. More notably, in the surgical cohort (Cases 5 and 14), recurrences were specifically localized to the surgical bronchial stump. Because these lesions were situated adjacent to the proximal lobar orifices, highly conservative resection margins were intentionally employed to avoid more extensive procedures (e.g., pneumonectomy or sleeve resections) for a benign disease, which inadvertently left microscopic residual elements. Management of these stump and local recurrences was successfully handled through repeat minimally invasive interventions or close surveillance.

### 3.4. Landscape of the Literature Review

A comprehensive review of the literature published between 2013 and 2025 identified 31 eligible clinical studies (Table 4). The aggregated data highlights a diverse therapeutic landscape for the management of endobronchial hamartomas, illustrating a distinct paradigm shift toward minimally invasive interventions over the past decade. All 31 included studies were appraised using the JBI critical appraisal checklist. Overall, the methodological quality was acceptable. Most studies provided clear information on patient characteristics, diagnosis, intervention, and outcomes. Some studies lacked detailed reporting of adverse events or long-term follow-up. No studies were found to have critical methodological flaws that would invalidate the synthesis.

### 3.5. Distribution of Therapeutic Modalities

Based on the extracted literature, interventional pulmonology-based approaches predominated among the reported therapeutic strategies. Bronchoscopic modalities represented the primary treatment approach in most cases, employing a variety of techniques, including flexible or rigid bronchoscopy with electrosurgical snares, argon plasma coagulation (APC), cryotherapy, and Nd:YAG laser ablation. Conversely, definitive surgical interventions were reserved for specific anatomical challenges but demonstrated a trend toward lung-sparing techniques, including segmentectomy [14], uniportal VATS wedge resection [20], and robotic-assisted sleeve resection [15]. Furthermore, complex or anatomically challenging lesions occasionally necessitated pre-planned multimodal strategies, such as combined laser ablation and lobectomy [22], while conservative management with regular surveillance was deemed appropriate in one asymptomatic case [17].

A structured synthesis of the 31 extracted studies further reveals distinct treatment patterns based on airway accessibility. Flexible bronchoscopy, often coupled with electrosurgical snares or cryotherapy, was the overwhelmingly preferred modality for distal or pedunculated lesions (*n* = 15 studies). Conversely, rigid bronchoscopy—frequently utilizing laser ablation or mechanical coring—was primarily reserved for proximal, highly obstructive lesions within the mainstem trachea or primary bronchi (*n* = 5 studies). Surgical interventions, while less frequently reported as primary therapies in the contemporary era, were systematically utilized as salvage procedures or for lesions deeply infiltrating the bronchial wall where endoscopic clearance was structurally compromised. This structured literature landscape perfectly mirrors the clinical decision-making algorithm observed in our 17-patient institutional cohort.

### 3.6. Peri-Procedural Safety and Complications

Overall, the available literature suggests a favorable perioperative safety profile for the reported therapeutic interventions. Most included studies reported no major procedure-related complications. Notably, no severe adverse events, life-threatening hemorrhage, or procedure-related mortality were described, indicating that contemporary bronchoscopic and selected surgical approaches are generally safe and feasible.

### 3.7. Follow-Up Outcomes and Recurrence Rate

Recurrence was infrequently reported in the reviewed literature. Most studies described no evidence of recurrence after treatment, and only one case series by Aktaş et al. reported local recurrence, in which one patient experienced lesion regrowth [24]. Overall, these findings suggest that the currently adopted interventional approaches provide satisfactory local disease control in most reported cases.

## 4. Discussion

Integrating our single-center experience with the systematically pooled contemporary literature reveals that while both bronchoscopic resection and surgical intervention maintain favorable safety profiles, their complication signatures are distinct. For EBH—a pathologically benign entity—the primary clinical paradigm has shifted from a binary assessment of “resectability” to achieving an optimal equilibrium between the complete relief of airway obstruction and the mitigation of iatrogenic trauma. Consequently, perioperative morbidity and the success rate of lung parenchyma preservation have emerged as the pivotal metrics for selecting between interventional and surgical modalities.

Data from our cohort and the summarized literature indicate that bronchoscopic resection is associated with a remarkably low overall complication rate. These adverse events are primarily localized and procedure-related, such as minor intraoperative or postoperative oozing, transient mucosal injury, and rare instances of perforation or localized pneumothorax secondary to thermal energy application [24,42]. As highlighted by the seminal retrospective study by Kim et al., procedure-related mortality in bronchoscopic management is nearly negligible, with serious long-term complications being exceedingly rare [43]. Our findings substantiate this; for patients with intraluminal lesions and narrow bases, bronchoscopy offers superior safety and a profound minimally invasive advantage. Collectively, these minor complications are typically manageable through immediate endoscopic intervention, thereby significantly reducing perioperative morbidity and maximizing the success of parenchyma preservation.

Conversely, surgical intervention remains an indispensable cornerstone when the tumor base is broad, transmural extension is evident, or the distal lung parenchyma has undergone irreversible destruction due to chronic obstruction. Although surgical resection ensures maximal local control and minimizes recurrence, it entails a more complex complication profile. Seminal research by Cosío et al. and recent systematic reviews consistently indicate that surgical cohorts exhibit significantly higher morbidity compared to interventional groups [44]. For patients undergoing segmentectomy or lobectomy, perioperative risks extend beyond wound infections, pleural effusions, and prolonged air leaks; the more profound impact is the permanent loss of healthy lung parenchyma and the concomitant decline in pulmonary functional reserve. For an essentially benign tumor, the potential for permanent functional impairment remains a central consideration in clinical decision-making.

Therefore, our synthesis of the literature and clinical data suggests that the disparity in complication profiles reflects a fundamental trade-off between minimally invasive intervention and traditional surgery. Rather than being competing modalities, they constitute a complementary therapeutic hierarchy. Clinicians should utilize high-resolution CT and bronchoscopy to meticulously evaluate the tumor base and distal lung salvageability. A “bronchoscopy-first” strategy should be prioritized for eligible cases to minimize morbidity, reserving surgical resection—and its associated perioperative risks—for anatomically complex or advanced cases.

Surgical intervention maintains a definitive advantage in long-term localized control. Both our study and the existing literature demonstrate that complete surgical resection yields an exceptionally low recurrence rate, effectively reducing the re-intervention rate to nearly zero. This “one-stop” therapeutic model provides patients with the highest degree of curative certainty, permanently resolving the risk of recurrent airway obstruction. However, our cohort distinctly highlights an important surgical caveat: recurrence post-lobectomy is not completely eliminated (as seen in Cases 5 and 14). When EBHs are located proximally near lobar bifurcations and exhibit deep transmural invasion, thoracic surgeons are forced into a difficult compromise. To prioritize the preservation of adjacent healthy lobes and avoid excessive morbidity (such as pneumonectomy), surgeons may accept a microscopically close or positive margin at the bronchial stump. This conservative, lung-sparing margin is the primary genesis of post-surgical stump recurrence, reaffirming that the biological behavior of EBH—specifically its deep mesenchymal root—can challenge both endoscopic and surgical boundaries. However, as previously discussed, this high level of oncological control is often achieved at the expense of higher perioperative morbidity and irreversible loss of lung function.

In contrast, while bronchoscopic resection maximizes parenchyma preservation, it is associated with higher rates of recurrence and re-intervention compared to surgery. This does not signify a failure of interventional technology, but rather reflects the intrinsic pathological characteristics of EBH and the physical limitations of endoscopic access. Research by Aktaş et al. noted that when a tumor is broad-based, densely adherent to the bronchial wall, or involves mesenchymal components extending beyond the cartilaginous ring, endoscopic snare or thermal ablation may struggle to completely eradicate deep-seated root tissues [24]. These residual components may proliferate slowly over time, eventually manifesting as clinical recurrence.

Furthermore, it is crucial to recognize that a 3-month follow-up—while standard for assessing immediate mucosal healing and residual regrowth—is undeniably insufficient to capture the full spectrum of late EBH recurrence. For example, Case 5 in our cohort demonstrated a delayed relapse 12 months post-surgery. To mitigate the limitations of our short institutional follow-up, the integrated systematic review serves as a vital complementary pillar. By aggregating contemporary literature with prolonged surveillance periods (up to 36 months), we can confidently infer that while early procedural success is consistently high across modalities, the longitudinal risk of indolent regrowth necessitates persistent surveillance beyond the initial 3-month healing phase.

It is essential to critically analyze the “re-intervention rate” associated with bronchoscopy. EBH is an indolent, slow-growing benign lesion; even in cases of in situ recurrence, the majority of patients can be effectively managed through repeat minimally invasive procedures such as electrocautery, snaring, cryoablation, or argon plasma coagulation. Essentially, clinicians are trading a manageable re-intervention rate for the substantial long-term benefits of avoiding thoracotomy and permanent pulmonary impairment. This strategy of managing a benign airway tumor as a “chronic condition” through minimally invasive means often proves superior in terms of both health economics and patient quality of life.

Notably, the recurrence rate in our cohort (17.6%, 3/17) appears higher than the aggregated rate observed in our systematic review. This discrepancy is likely attributable to two factors. First, the limited sample size of our cohort means that a small absolute number of recurrences results in a disproportionately high percentage, which is highly susceptible to small-sample statistical variance. Second, the existing literature—predominantly comprising case reports—is inherently subject to publication bias. Successful, single-session interventions are preferentially reported, whereas cases complicated by recurrence are likely underreported, thereby artificially lowering the perceived recurrence rate in the published literature.

Nonetheless, the inherently low recurrence risk following traditional surgery does not justify the neglect of long-term surveillance. Given the rarity of EBH and the potential for residual disease or postoperative airway remodeling, regular follow-up remains mandatory. For surgical patients, periodic radiological imaging—supplemented by bronchoscopy when necessary—is required to monitor for rare recurrences. For patients treated endoscopically, a standardized and prolonged follow-up regimen is even more critical to ensuring long-term airway patency and clinical stability.

## 5. Limitations

Several limitations inherent to this study design must be acknowledged. Primarily, the constrained cohort size—a direct consequence of the extreme epidemiological rarity of EBH—precludes extensive subgroup analyses and may limit the broader statistical power of our findings. Secondly, the median follow-up time of our institutional cohort is relatively short (3–36 months). While this timeframe is the literature standard for assessing early mucosal healing and procedural thoroughness, it limits the statistical power to independently characterize the long-term recurrence trajectory. However, this limitation is effectively offset by our dual-design approach; the inclusion of the systematic review, encompassing studies with multi-year follow-ups, successfully bridges this temporal gap and provides a more comprehensive long-term prognostic landscape. Thirdly, we observed an epidemiological deviation in our cohort regarding gender distribution. While historical literature suggests a strong male predominance (2–4 times higher) for EBH, our cohort exhibited a slight female majority (58.8%). We hypothesize this is primarily a statistical artifact secondary to our small sample size, where minor variations can disproportionately skew demographic ratios. Alternatively, it may reflect evolving diagnostic patterns; the contemporary widespread use of high-resolution computed tomography (HRCT) for routine health screenings may be identifying more asymptomatic or early-stage EBHs in female patients than in historical cohorts. However, larger, multi-center registries are required to validate whether a true epidemiological shift is occurring. Additionally, the retrospective nature of the data collection introduces potential selection and information biases. While our institutional experience strongly aligns with the synthesized literature, the establishment of prospective, multicenter clinical registries is ultimately warranted to validate these therapeutic algorithms and definitively elucidate long-term recurrence patterns across diverse populations.

## 6. Implications for Clinical Practice and Future Research

The findings of this study have important implications for the clinical management of endobronchial hamartoma (EBH). Given the excellent safety profile and superior lung parenchyma-preserving capacity of interventional bronchoscopy, we recommend that it be adopted as the first-line treatment for all eligible patients with intraluminal, pedunculated, or small broad-based lesions. Surgical resection should be strictly reserved for anatomically complex lesions with transmural extension, or cases where irreversible distal parenchymal destruction has already occurred. Notably, since local recurrence can occur even after definitive anatomical surgery, all patients, regardless of the treatment modality received, should undergo regular and long-term clinical and bronchoscopic surveillance.

From a health system perspective, the establishment of a national or international multicenter registry for rare airway tumors, including EBH, is urgently needed. Such a registry would facilitate the collection of large-scale, standardized clinical data, which is essential for addressing the current evidence gaps in this field.

Future research should focus on three key areas: (1) prospective comparative studies to evaluate the long-term efficacy and safety of different bronchoscopic techniques (e.g., snare resection, cryotherapy, laser ablation) for EBH; (2) identification of clinical and pathological predictors of recurrence to guide individualized surveillance strategies; and (3) development of standardized diagnostic and therapeutic algorithms for EBH to improve clinical decision-making worldwide.

## 7. Conclusions

Endobronchial hamartoma is an exceptionally rare benign neoplasm of the central airways that necessitates rigorous histopathological confirmation. Our findings, supported by a comprehensive review of the current literature, suggest a potential clinical paradigm shift toward lung-sparing, minimally invasive strategies. Interventional bronchoscopy may be considered a preferable initial treatment option for anatomically suitable EBHs, offering advantages in minimizing iatrogenic trauma and preserving pulmonary functional reserve. Anatomical surgical resection, while highly effective for local disease control, should be strictly reserved for anatomically complex, broad-based lesions or instances involving irreversible distal parenchymal destruction. Given the lower level of evidence inherent to retrospective cohorts and the documented potential for insidious local recurrence across all modalities, these conclusions should be interpreted with caution. Ultimately, vigilant and longitudinal surveillance remains an indispensable component in the holistic management of EBH.

## Figures and Tables

**Table 1:** Baseline demographic and clinical characteristics of patients with endobronchial hamartoma (*N* = 17).

Case	Age (y)	Sex	Smoking History	Medica History	Chief Complaint(s)	Symptom Duration
1	65	Female	Never	Post-appendectomy	Exertional dyspnea	15 days
2	51	Female	Never	Bronchial asthma	Asymptomatic	-
3	43	Female	Never	Rib fracture	Cough and chest pain	1 month
4	53	Male	Never	None	Right back pain	2 months
5	54	Female	Never	Nodular goiter	Chest pain	1 year
6	36	Female	Never	Pharyngitis	Cough and fever	15 days
7	63	Female	Never	Hypertension	Cough and expectoration	7 days
8	28	Male	Never	None	Asymptomatic	1 month
9	73	Male	Never	Hypertension	Cough and dyspnea	1 month
10	68	Female	Never	None	Cough and chest tightness	1 month
11	56	Male	Current/Former	None	Cough and expectoration	1 month
12	58	Female	Never	None	Cough and expectoration	14 days
13	26	Male	Never	None	Cough, expectoration, and chest pain	2 months
14	69	Male	Never	None	Cough and chest tightness	1 month
15	79	Female	Never	Arrhythmia	Cough and expectoration	10 years
16	88	Male	Never	None	Cough and expectoration	1 month
17	68	Female	Never	None	Cough and expectoration	3 months

**Table 2:** Radiological and bronchoscopic characteristics of endobronchial hamartomas.

Case	Tumor Location	Size (cm)	CT Features	Secondary Radiological Signs	Bronchoscopic Appearance
1	RML bronchus	1.3 × 0.9	Non-specific	Obstructive pneumonia (RML)	Smooth surface, pedunculated
2	RLL basal segment	1.0 × 0.5	Non-specific	None	Smooth surface
3	Left main bronchus	0.7 × 0.6	Non-specific	Inflammatory nodule (RLL)	Smooth surface, pedunculated
4	RUL posterior segment	0.3 × 0.2, 0.5 × 0.4	Non-specific	None	Smooth surface
5	RUL bronchus	1.6 × 1.6	Non-specific	None	Non-specific
6	RLL posterior basal segment	2.0 × 1.4	Non-specific	Pulmonary pneumatocele (RLL)	Smooth surface, pedunculated
7	RUL bronchus	2.0 × 1.0	Non-specific	None	Non-specific
8	RLL bronchus	2.0 × 1.5, 3.0 × 2.5	Non-specific	None	Non-specific
9	LLL bronchus	1.3 × 1.3	Non-specific	None	Non-specific
10	LLL bronchus	3.0 × 2.6	Non-specific	None	Non-specific
11	RLL bronchus	0.8 × 0.5	Non-specific	Pulmonary infection	Non-specific
12	RML bronchus	2.1 × 1.9	Non-specific	None	Smooth surface
13	Right main bronchus	0.8 × 0.4	Non-specific	None	Cauliflower-like neoplasm
14	LLL bronchus	0.5 × 0.4	Non-specific	None	Non-specific
15	RML bronchus	1.9 × 1.7	Non-specific	Pleural effusion	Non-specific
16	RLL bronchus	7.5 × 5.4	Non-specific	Pneumothorax	Non-specific
17	LUL bronchus	2.3 × 1.8	Non-specific	Pneumothorax	Non-specific

**Table 3:** Therapeutic interventions, pathological findings, and clinical outcomes.

Case No.	Primary Intervention	Intraoperative Bleeding	Pathological Confirmation	Follow-Up	Recurrence
1	Bronchoscopic electrosurgical snare + cryoablation	Minimal	Hamartoma (cartilage, adipose tissue)	3 months	Yes
2	Bronchoscopic electrosurgical snare + cryoablation	Minimal	Chondroid hamartoma	12 months	No
3	Bronchoscopic electrosurgical snare	Minimal	Hamartoma (mature mucous glands, cartilage, fat)	5 months	No
4	Bronchoscopic electrosurgical snare + cryoablation	Minimal	Hamartoma (cartilage, adipose tissue)	6 months	No
5	Lobectomy	Minimal	Hamartoma (cartilage, adipose tissue)	12 months	Yes
6	Bronchoscopic electrosurgical snare	Scant	Chondroid hamartoma	12 months	No
7	Percutaneous needle biopsy + Bronchoscopic electrosurgical snare	Minimal	Chondroid hamartoma	12 months	No
8	Mass enucleation (RLL)	Scant	Chondroid hamartoma	3 months	No
9	Percutaneous needle biopsy+ Lobectomy	Minimal	Hamartoma (ciliated columnar epithelium, stromal myxoid change, adipose vacuoles)	3 months	No
10	Lobectomy	Scant	Hamartoma (smooth muscle component)	3 months	No
11	Bronchoscopic electrosurgical snare	Minimal	Chondroid hamartoma	3 months	No
12	Bronchoscopic electrosurgical snare	Minimal	Chondroid hamartoma	3 months	No
13	Bronchoscopic electrosurgical snare	Minimal	Hamartoma (cartilage, adipose tissue)	3 months	No
14	Lobectomy	Scant	Hamartoma (adipose tissue)	3 months	Yes
15	Percutaneous needle biopsy + Bronchoscopic electrosurgical snare	Minimal	Hamartoma (ciliated columnar epithelium, smooth muscle with myxoid degeneration)	3 months	No
16	Percutaneous needle biopsy + Bronchoscopic electrosurgical snare	Minimal	Hamartoma (cartilage and adipose tissue)	36 months	No
17	Percutaneous needle biopsy + Lobectomy	Minimal	Hamartoma (adipose tissue)	3 months	No

**Table 4:** Summary of literature review on endobronchial interventions and clinical outcomes. Abbreviation: NR (None report).

Author(s), Year	Study Design	Primary Therapeutic Modality (Approach and Tool)	Major Complications	Follow-Up	Recurrence
Peng et al., 2025 [1]	Case report	Rigid bronchoscopic electrosurgical snare	None	NR	None
Touil et al., 2025 [12]	Case series	Rigid bronchoscopic laser ablation	None	12–36 months	None
Chopra et al., 2025 [13]	Case series	Flexible bronchoscopic electrocautery ± cryotherapy	None	12–18 months	None
Daboussi et al., 2025 [14]	Case report	Surgical segmentectomy	NR	NR	None
Igai et al., 2025 [15]	Case report	Robotic-assisted sleeve resection (intermediate bronchus)	NR	NR	NR
Cano et al., 2024 [16]	Case report	Bronchoscopic resection (unspecified)	NR	NR	NR
Bouanani et al., 2024 [17]	Case report	Conservative management (Regular follow-up)	None	NR	None
Cesaro et al., 2025 [18]	Case series	Combined flexible (laser) and rigid bronchoscopy	None	3–36 months	None
Fernandez-Trujillo et al., 2023 [19]	Case report	Bronchoscopic electrocautery with cryotherapy	None	6 months	None
Jiao et al., 2023 [20]	Case report	Uniportal VATS bronchial wedge resection	None	6 months	None
Ng et al., 2020 [21]	Case report	Flexible bronchoscopic cryoprobe extraction	None	3 months	None
Caterino et al., 2020 [22]	Case report	Multimodal: Rigid bronchoscopy (YAG laser) + lobectomy	NR	NR	NR
Cherian et al., 2019 [23]	Case report	Bronchoscopic argon plasma coagulation (APC)	None	2 months	None
Aktaş et al., 2018 [24]	Case series	Interventional bronchoscopic therapy (standard)	None	36.0 ± 15.0 m	1 (4.8%)
Triviño et al., 2019 [25]	Case report	Rigid bronchoscopy (ligasure technique)	None	6 months	None
Chen et al., 2017 [26]	Case report	Multimodal: Bronchoscopy + systemic chemotherapy	NR	NR	NR
Liu et al., 2017 [27]	Case report	Bronchoscopic snare electrocautery	None	12 months	None
Omar et al., 2016 [28]	Case report	Interventional bronchoscopy (snare + APC)	None	3 months	None
Lococo et al., 2016 [29]	Case report	Bronchoscopic snare electrocautery	None	12 months	None
Kim et al., 2016 [30]	Case report	Bronchoscopic cryotherapy	NR	NR	NR
Connell et al.,2015 [31]	Case report	Flexible bronchoscopy (snare + APC)	None	2 months	None
Kim et al., 2015 [32]	Case report	Flexible bronchoscopy (electrosurgical snare)	None	3 months	None
Scarlata et al., 2015 [33]	Case series	Rigid bronchoscopic tracheobronchial resection	None	NR	NR
Mertoğlu et al., 2017 [34]	Case report	Bronchoscopic APC and electrocoagulation	None	24 months	None
Ucar et al., 2014 [35]	Case report	Bronchoscopic snare-cryoextraction sequence	None	24 months	None
Celik et al., 2014 [36]	Case series	Surgical resection (lobectomy/bronchotomy)	None	NR	None
Sarioglu et al., 2014 [37]	Case report	Flexible bronchoscopy (unspecified)	None	1 month	None
Sim et al., 2014 [38]	Case report	Bronchoscopic cryotherapy	None	1–3 months	None
El-Kersh et al., 2014 [39]	Case report	Bronchoscopic piecemeal snare + APC	None	1 month	None
Poonja et al., 2013 [40]	Case report	Bronchoscopic snare electrocautery	None	NR	None
Miller et al., 2013 [41]	Case series	Bronchoscopic APC with electrocautery/cryotherapy	None	6 months	None

## Data Availability

Restrictions apply to the availability of data generated or analyzed during this study to preserve patient confidentiality or because they were used under license. Data are, however, available from the authors upon reasonable request.

## Supplementary Materials

The following supporting information can be downloaded at: https://www.mdpi.com/article/10.3390/jcm15103616/s1, File S1: PRISMA Checklist. [45].
