# Supplementary material for: Endobronchial Hamartoma: A Retrospective Cohort Study of 17 Cases and Systematic Review of the Contemporary Literature
Source: J Clin Med. 2026 May 8;15(10):3616. doi: 10.3390/jcm15103616 (PMC13207699; doi:10.3390/jcm15103616)
Supplement: Supplementary file 1 [file jcm-15-03616-s001.zip › PRISMA_2020_checklist.pdf]

PRISMA 2020 Checklist

Section and Topic	Item #	Checklist item	Location where item is reported
**TITLE**			
Title	1	Identify the report as a systematic review.	Line 1-2
**ABSTRACT**			
Abstract	2	See the PRISMA 2020 for Abstracts checklist.	Line 12-43
**INTRODUCTION**			
Rationale	3	Describe the rationale for the review in the context of existing knowledge.	Line 46-73
Objectives	4	Provide an explicit statement of the objective(s) or question(s) the review addresses.	Line 73-84
**METHODS**			
Eligibility criteria	5	Specify the inclusion and exclusion criteria for the review and how studies were grouped for the syntheses.	Line 87-101, Line 114-122
Information sources	6	Specify all databases, registers, websites, organisations, reference lists and other sources searched or consulted to identify studies. Specify the date when each source was last searched or consulted.	Line 114-122
Search strategy	7	Present the full search strategies for all databases, registers and websites, including any filters and limits used.	Line 114-122
Selection process	8	Specify the methods used to decide whether a study met the inclusion criteria of the review, including how many reviewers screened each record and each report retrieved, whether they worked independently, and if applicable, details of automation tools used in the process.	Line 114-122
Data collection process	9	Specify the methods used to collect data from reports, including how many reviewers collected data from each report, whether they worked independently, any processes for obtaining or confirming data from study investigators, and if applicable, details of automation tools used in the process.	Line 114-122
Data items	10a	List and define all outcomes for which data were sought. Specify whether all results that were compatible with each outcome domain in each study were sought (e.g. for all measures, time points, analyses), and if not, the methods used to decide which results to collect.	Line 114-122
	10b	List and define all other variables for which data were sought (e.g. participant and intervention characteristics, funding sources). Describe any assumptions made about any missing or unclear information.	Line 114-122
Study risk of bias assessment	11	Specify the methods used to assess risk of bias in the included studies, including details of the tool(s) used, how many reviewers assessed each study and whether they worked independently, and if applicable, details of automation tools used in the process.	Line 133-144
Effect measures	12	Specify for each outcome the effect measure(s) (e.g. risk ratio, mean difference) used in the synthesis or presentation of results.	Line 103-112
Synthesis methods	13a	Describe the processes used to decide which studies were eligible for each synthesis (e.g. tabulating the study intervention characteristics and comparing against the planned groups for each synthesis (item #5)).	Line 124-131
	13b	Describe any methods required to prepare the data for presentation or synthesis, such as handling of missing summary statistics, or data conversions.	Line 124-131
	13c	Describe any methods used to tabulate or visually display results of individual studies and syntheses.	Line 206-238
	13d	Describe any methods used to synthesize results and provide a rationale for the choice(s). If meta-analysis was performed, describe the model(s), method(s) to identify the presence and extent of statistical heterogeneity, and software package(s) used.	Results → 3.4-3.7 + Discussion: Qualitative descriptive synthesis method was adopted without conducting

PRISMA 2020 Checklist

Section and Topic	Item #	Checklist item	Location where item is reported
			meta-analysis. Reason: Most of the included studies were case reports with significant heterogeneity, lacking quantifiable data that could be pooled. Technical success rates, complication rates, recurrence rates, and lung preservation for different treatment methods were summarized, and a comprehensive analysis was conducted in conjunction with cohort data from 17 cases in our center
	13e	Describe any methods used to explore possible causes of heterogeneity among study results (e.g. subgroup analysis, meta-regression).	No statistical heterogeneity analysis or subgroup analysis was conducted (no quantitative pooled data); qualitative exploration of clinical heterogeneity was performed based on three dimensions: treatment method, lesion morphology, and

Section and Topic	Item #	Checklist item	Location where item is reported
			follow-up duration, and the impact of these factors on treatment outcomes was discussed in the results section.
	13f	Describe any sensitivity analyses conducted to assess robustness of the synthesized results.	Not conducted. Reason: The included studies were all case reports and small-sample case series, which only underwent qualitative descriptive synthesis. There was no quantifiable data to combine, making sensitivity analysis inapplicable.
Reporting bias assessment	14	Describe any methods used to assess risk of bias due to missing results in a synthesis (arising from reporting biases).	Not conducted. Reason: Without quantitative meta-analysis, formal publication bias assessments such as funnel plots cannot be performed; the potential impact of publication bias has been qualitatively discussed in the discussion section.
Certainty assessment	15	Describe any methods used to assess certainty (or confidence) in the body of evidence for an outcome.	Not conducted. Reason: The

PRISMA 2020 Checklist

Section and Topic	Item #	Checklist item	Location where item is reported
			GRADE framework is primarily applicable to quantitative synthesis of intervention studies. This study is a qualitative systematic review of rare diseases, and thus, it is not suitable for evidence grading.
**RESULTS**			
Study selection	16a	Describe the results of the search and selection process, from the number of records identified in the search to the number of studies included in the review, ideally using a flow diagram.	Figure 2
	16b	Cite studies that might appear to meet the inclusion criteria, but which were excluded, and explain why they were excluded.	Figure 2
Study characteristics	17	Cite each included study and present its characteristics.	Line 217-221<br>Table 4
Risk of bias in studies	18	Present assessments of risk of bias for each included study.	Line 222-226
Results of individual studies	19	For all outcomes, present, for each study: (a) summary statistics for each group (where appropriate) and (b) an effect estimate and its precision (e.g. confidence/credible interval), ideally using structured tables or plots.	Line 217-249
Results of syntheses	20a	For each synthesis, briefly summarise the characteristics and risk of bias among contributing studies.	Line 222-226
	20b	Present results of all statistical syntheses conducted. If meta-analysis was done, present for each the summary estimate and its precision (e.g. confidence/credible interval) and measures of statistical heterogeneity. If comparing groups, describe the direction of the effect.	Line 228-254
	20c	Present results of all investigations of possible causes of heterogeneity among study results.	Line 228-254
	20d	Present results of all sensitivity analyses conducted to assess the robustness of the synthesized results.	Line 228-254
Reporting biases	21	Present assessments of risk of bias due to missing results (arising from reporting biases) for each synthesis assessed.	Line 228-254
Certainty of evidence	22	Present assessments of certainty (or confidence) in the body of evidence for each outcome assessed.	Line 228-254
**DISCUSSION**			
Discussion	23a	Provide a general interpretation of the results in the context of other evidence.	Line 257-309
	23b	Discuss any limitations of the evidence included in the review.	Line 339-348
	23c	Discuss any limitations of the review processes used.	Line 339-348
	23d	Discuss implications of the results for practice, policy, and future research.	Line 350-371

PRISMA 2020 Checklist

Section and Topic	Item #	Checklist item	Location where item is reported
**OTHER INFORMATION**			
Registration and protocol	24a	Provide registration information for the review, including register name and registration number, or state that the review was not registered.	Methodology → Study Population and Data Collection: The cohort part was registered on ClinicalTrials.gov (NCT06082791), while the systematic review was not separately registered
	24b	Indicate where the review protocol can be accessed, or state that a protocol was not prepared.	Methods, Section 2.1. No public study protocol was developed for this study.
	24c	Describe and explain any amendments to information provided at registration or in the protocol.	Methods, Section 2.1. No amendments were made to the registration information or study protocol during the study period.
Support	25	Describe sources of financial or non-financial support for the review, and the role of the funders or sponsors in the review.	Funding section at the end of the manuscript. Four funding sources are listed, and it is stated that the funders had no role in the study design, data collection and analysis, manuscript writing, or submission decision.
Competing	26	Declare any competing interests of review authors.	Competing

PRISMA 2020 Checklist

Section and Topic	Item #	Checklist item	Location where item is reported
interests			Interests section at the end of the manuscript. The authors declare no competing interests.
Availability of data, code and other materials	27	Report which of the following are publicly available and where they can be found: template data collection forms; data extracted from included studies; data used for all analyses; analytic code; any other materials used in the review.	Data Availability section at the end of the manuscript. Raw data are available upon reasonable request from the corresponding author; no analytic code or other standardized research materials were generated for this study.

From: Page MJ, McKenzie JE, Bossuyt PM, Boutron I, Hoffmann TC, Mulrow CD, et al. The PRISMA 2020 statement: an updated guideline for reporting systematic reviews. BMJ 2021;372:n71. doi: 10.1136/bmj.n71. This work is licensed under CC BY 4.0. To view a copy of this license, visit https://creativecommons.org/licenses/by/4.0/
